# Long Non-Coding RNA Encoded by Infectious Bronchitis Virus Facilitates Viral Replication via Direct Interaction with G3BP2 and Expression Regulation of a Novel Host MicroRNA

**DOI:** 10.3390/vetsci13030215

**Published:** 2026-02-25

**Authors:** Mingjing Zhang, Zhichao Cai, Hongliu An, Rong He, Songbai Zhang, Shouguo Fang

**Affiliations:** College of Agriculture, Yangtze University, Jingzhou 434025, China; echozh@hotmail.com (M.Z.); caizhichao110@126.com (Z.C.); 1984625825@163.com (H.A.); coco-hey@outlook.com (R.H.)

**Keywords:** infectious bronchitis virus, long non-coding RNA, ras–GAP SH3 domain binding protein 2, novel miRNA (novel-340)

## Abstract

Long non-coding RNAs (lncRNAs) encoded by viruses play crucial roles in viral infection, pathogenesis processes, the interaction between viruses and hosts, and immune escape. Herein, by employing RNA pull-down, mass spectrometry technology, and RNA immunoprecipitation, we identified a host protein (G3BP2) that specifically interacts with the lncRNA encoded by the infectious bronchitis virus (IBV). Additionally, we identified a novel host miRNA (novel-340) in IBV-infected H1299 cells and further verified that novel-340 can target the 3′-UTR (untranslated region) of G3BP2 and downregulate its expression in a dose-dependent manner. We discovered that IBV-lncRNA may facilitate IBV replication in H1299 cells through direct interaction with G3BP2 and/or the regulation of the IBV-lncRNA/novel-340/G3BP2 interactive regulatory network. This work deepens the understanding of the biological function of IBV-lncRNA.

## Simple Summary

Virus-encoded long non-coding RNA and virus-induced host microRNA (miRNA) play significant roles in the viral life cycle. In this study, we identified a host protein, G3BP2, that specifically interacts with the long non-coding RNA encoded by the infectious bronchitis virus (IBV) (IBV-lncRNA) and a novel host miRNA (novel-340) that targets the 3′-untranslated region of G3BP2 in IBV-infected H1299 cells. We discovered that IBV-lncRNA can facilitate IBV replication through direct interaction with G3BP2 and regulation of the expression of novel-340. Our findings offer valuable insights into the biological functions of IBV-lncRNA.

## 1. Introduction

Virus-encoded long non-coding RNAs (lncRNAs) play critical roles in viral infection and pathogenesis, modulation of virus–host interactions, and regulation of host immune responses largely mediated through RNA–DNA, RNA–RNA, and RNA–protein interactions [1]. For instance, polyadenylated nuclear RNA (PAN), a 1.2 kb lncRNA encoded by Kaposi’s sarcoma-associated herpesvirus (KSHV), regulates both viral and host gene expression by interacting with multiple host proteins [2,3]. Similarly, the β2.7 lncRNA encoded by human cytomegalovirus (HCMV) suppresses virus-induced apoptosis and sustains ATP production by specifically binding to the key components of mitochondrial complex I, which is essential for efficient viral replication [4]. For flaviviruses, including dengue virus, yellow fever virus, West Nile virus, and Japanese encephalitis virus, subgenomic flaviviral RNA (sfRNA), a stable non-coding RNA fragment (~300–500 nt) generated via incomplete degradation of the viral 3′-untranslated region (3′-UTR) by the host 5′-3′ exoribonuclease XRN1 [5], functions as a key modulator of viral infectivity and host antiviral immunity [6].

MicroRNA (miRNA), typically ranging from 17 to 24 nt in length, belongs to the short non-coding RNA family [7]. miRNAs predominantly bind to the 3′-UTR of target gene mRNAs and play a pivotal role in gene expression regulation and translational repression [8]. Host miRNAs play a pivotal role in the virus–host interaction process. Their functions encompass the entire spectrum from virus adsorption to proliferation and replication. These miRNAs exert a bidirectional regulatory influence on virus infection via specific regulatory pathways; they can either impede infection or facilitate virus replication [9]. Their modes of action involve either directly binding to the viral genome or indirectly affecting the infection process by targeting and regulating host factors associated with virus replication [10]. Some host miRNAs are utilized by viruses to augment their own replication and infection capabilities. These miRNAs with proviral effects can aid viruses in evading the host immune response by suppressing antiviral factors such as interferons [9]. Conversely, some miRNAs perform antiviral functions, including activating antiviral mechanisms, inducing the virus to enter a latent state, or obstructing the infection process by directly inhibiting virus replication, downregulating viral protein expression, and targeting host mRNAs encoding proviral factors [11].

Infectious bronchitis virus (IBV), a member of the genus *Gammacoronavirus* within the family Coronaviridae, is an enveloped, positive-sense, single-stranded RNA virus [12]. We first reported the expression of a virus-derived long non-coding RNA (lncRNA) in IBV-infected cells. This lncRNA, designated IBV-lncRNA, comprises 563 nucleotides (excluding the poly(A) tail) and is derived from the fusion of the viral 3′-UTR and the 5′-leader sequence in the genome via discontinuous transcription [13]. However, the precise biological functions of IBV-lncRNA remain poorly characterized. To address this knowledge gap, the present study systematically investigated the functional role of IBV-lncRNA during IBV infection. However, the multifaceted roles of IBV-encoded lncRNAs, particularly their coordinated regulation of host factors such as G3BP2 and associated non-coding RNAs, remain poorly understood. Therefore, this study aims to systematically investigate the mechanisms by which IBV-lncRNAs modulate G3BP2 expression and function, and to explore their broader impact on the virus–host interaction network during infectious bronchitis virus infection.

## 2. Materials and Methods

### 2.1. Cell Culture and Virus Titration

The human non-small cell lung carcinoma cell line H1299 (CSTR: 19375.09.3101HUMTCHu160, purchased from the Cell Bank of the Committee on Type Culture Collection of Chinese Academy of Sciences) was maintained in RPMI-1640 medium supplemented with 10% fetal bovine serum (FBS). Vero cells, derived from African green monkey kidney (ATCC: CCL-81), were cultured in Dulbecco’s Modified Eagle Medium (DMEM) containing 10% FBS (RPMI-1640, DMEM, FBS, Gibco, Grand Island, NY, USA). All cell lines were incubated at 37 °C under 5% CO_2_.

The wild-type recombinant infectious bronchitis virus (rIBV), a Vero cell-adapted strain (IBV Beaudette p65; GenBank: DQ001339.1), was generated using reverse genetics and produces a noncoding RNA (ncRNA). The isogenic mutant strain, rIBV-C27107G, was engineered from the parental IBV-p65 backbone via site-directed mutagenesis and does not produce ncRNA [13]. Note: “ncRNA” herein specifically refers to the long non-coding RNA encoded by IBV, designated IBV-lncRNA.

Virus stocks were generated by infecting cells at a multiplicity of infection (MOI) of 0.1 in serum-free DMEM for 24–36 h until 80% cytopathic effect (CPE) was observed. The virus-containing supernatant was harvested, subjected to three freeze–thaw cycles, and centrifuged at 4000× *g* for 10 min at 4 °C to remove cell debris. The clarified supernatant was aliquoted and stored at −80 °C.

Viral titers were determined by infecting Vero cell monolayers with serially diluted supernatants for 2 h. Cells were overlaid with 0.4% agarose-DMEM and incubated for 36–72 h. Plaques were stained with 0.2% crystal violet after formaldehyde fixation. Titers (PFU/mL) were calculated from triplicate experiments.

PFU/mL = (number of plaques × dilution factor)/volume of inoculum (mL)

### 2.2. Plasmid Construction and Cell Transfection

The complete coding sequence (CDS) of the G3BP2 gene (2466 bp) was amplified from H1299 cell cDNA with gene-specific primers. All primer sequences used are listed in Table 1. The oligonucleotides were commercially synthesized by Tsingke Biotechnology (Wuhan, China).

For transfection, Lipofectamine™ 3000 reagent (Invitrogen, Waltham, MA, USA) was used following the manufacturer’s protocol. Briefly, cells were seeded into 6-well plates and cultured in RPMI-1640 supplemented with 10% FBS at 37 °C under 5% CO_2_ until reaching 70–80% confluency. To form transfection complexes, plasmids, miRNA mimics, or siRNAs were diluted in 250 μL of Opti-MEM medium (Gibco, Grand Island, NY, USA) and incubated for 5 min at room temperature. Separately, Lipofectamine™ 3000 reagent was diluted in an equal volume of Opti-MEM and then combined with the nucleic acid mixture. The resulting mixture was incubated for 15 min at room temperature to allow complex formation. The complexes were added dropwise to the cell cultures. After 6 h of incubation, the medium was replaced with fresh complete growth medium.

Following transfection, cells were processed according to the specific experimental requirements and stored at −80 °C. For functional expression analysis, cells were harvested directly at 36 h post-transfection. For viral infection assays, cells were inoculated with the virus at 24 h post-transfection with plasmids, siRNAs, or miRNA mimics, and samples were collected at the designated time points. All primer sequences used in this study are listed in Table 2. The oligonucleotides were commercially synthesized by Tsingke Biotechnology (Wuhan, China).

### 2.3. RNA Pull-Down Assay and Mass Spectrometry Analysis

RNA pull-down coupled with liquid chromatography–tandem mass spectrometry (LC-MS/MS) was used to identify host cellular proteins that specifically interact with IBV-lncRNA. Biotin-labeled IBV-lncRNA was synthesized by in vitro transcription using the RiboMAX™ Large Scale RNA Production System-T7 kit (Promega, Madison, WI, USA). The biotinylated RNA was incubated with streptavidin magnetic beads to capture RNA-binding proteins through the high-affinity streptavidin-biotin interaction. After washing, bound proteins were eluted and analyzed by LC-MS/MS to identify potential interacting partners.

The protein sequences identified by mass spectrometry were filtered against the Homo sapiens protein sequences in the UniProt database (accessed on 22 March 2024 from website: https://www.uniprot.org/). Gene Ontology (GO) and Kyoto Encyclopedia of Genes and Genomes (KEGG) pathway enrichment analyses were then performed using the ClusterProfiler software (version 4.0, https://bioconductor.org/packages/clusterProfiler/) to systematically predict the potential biological functions of the target genes.

### 2.4. RNA Immunoprecipitation (RIP)

RNA immunoprecipitation (RIP) was performed using a commercial kit (FI8709, Guangzhou Huijun Biotech Co., Ltd., Guangzhou, China) to identify RNAs bound by specific host (bait) proteins. Cell lysates were incubated with an anti-FLAG antibody or a control IgG for 4 h. Protein A/G magnetic beads were then added, and incubation continued for an additional 4 h to capture the antigen–antibody complexes. The bead-bound complexes were washed, and a portion was eluted with 1× SDS-PAGE loading buffer for Western blot analysis to confirm immunoprecipitation specificity. RNA co-precipitated with the complexes was purified and analyzed by RT-qPCR to determine target RNA expression levels.

### 2.5. RNA Interference (RNAi) Assay

For RNA interference (RNAi) experiments, a gene-specific siRNA targeting G3BP2 was designed (primer sequences provided in Table 2; synthesized by Tsingke Biotechnology, Wuhan, China). Transfection experiments were performed using non-targeting control siRNA (NC siRNA) and GAPDH siRNA as controls. At 48 h post-transfection, the silencing efficiency of G3BP2-siRNA was evaluated by RT-qPCR and Western blot to identify effective siRNA sequences for subsequent studies.

The selected effective G3BP2-siRNA was transfected into H1299 cells to knock down G3BP2 protein expression. At 24 h after transfection, cells were infected with either rIBV-C27107G or wild-type rIBV at equivalent MOI. Total RNA and proteins were collected at 24 h post-infection and analyzed by RT-qPCR and Western blot to evaluate the expression levels of relevant genes.

### 2.6. Dual-Luciferase Reporter Assay

To predict potential binding sites between novel-340 and its target gene G3BP2, we used RNAHybrid (accessed on 15 July 2025 from the website: https://bibiserv.cebitec.uni-bielefeld.de/rnahybrid/RNAhybrid). Based on the predictions, wild-type and mutant DNA fragments (Table 3) were cloned into the pmirGLO vector to generate the pmirGLO-G3BP2-WT and pmirGLO-G3BP2-MUT reporter plasmids, which were synthesized by Tsingke Biotechnology.

To functionally validate the predicted interaction between novel-340 and G3BP2 (NM_012297.5), a dual-luciferase reporter assay was performed. Cells were co-transfected with either the wild-type reporter plasmid pmirGLO-G3BP2-3′UTR-WT or its mutant counterpart pmirGLO-G3BP2-3′UTR-MUT, together with novel-340 mimics or negative control (NC) mimics (synthesized by Tsingke Biotechnology, Wuhan, China). Luciferase activity was then measured using the Dual-Luciferase^®^ Reporter Assay System (DL101-01; Vazyme, Nanjing, China) according to the manufacturer’s protocol.

Cells were divided into the following four transfection groups, each performed in triplicate:

(a) NC mimics + pmirGLO-G3BP2-3′-UTR-WT
(b) novel-340 mimics + pmirGLO-G3BP2-3′-UTR-WT
(c) NC mimics + pmirGLO-G3BP2-3′-UTR-MUT
(d) novel-340 mimics + pmirGLO-G3BP2-3′-UTR-MUT

The final concentrations used were 50 nM for both NC mimics and novel-340 mimics, and 500 ng for both the pmirGLO-G3BP2-3′UTR-WT and pmirGLO-G3BP2-3′UTR-MUT plasmids in all transfection groups.

### 2.7. Quantitative Real-Time PCR (RT-qPCR)

Total RNA was extracted with TRIzol reagent (Invitrogen, Waltham, MA, USA). Then, 1.0 μg of RNA was reverse-transcribed into cDNA. RT-qPCR was performed using a SYBR Green-based kit (Takara Bio Inc., Shiga, Japan) according to the manufacturer’s instructions. The cycling conditions consisted of an initial denaturation at 95 °C for 3 min, followed by 40 cycles of 95 °C for 30 s, annealing for 1 min, and fluorescence acquisition at 72 °C for 1 min. Relative gene expression was normalized to GAPDH/U6 and calculated via the 2^−ΔΔCt^ method. The annealing temperatures for the primer sequences are provided in Table 4.

### 2.8. Western Blotting

Following treatment, cells were washed with PBS (phosphate-buffered saline) and lysed in RIPA (radioimmunoprecipitation assay) buffer containing PMSF (phenylmethanesulfonyl fluoride) (Thermo Fisher Scientific, Waltham, MA, USA). The lysates were centrifuged, and the supernatants were combined with 5× SDS loading buffer and denatured at 100 °C for 10 min. Proteins were separated by 10% SDS-PAGE and transferred to PVDF membranes (Stratagene San Diego, CA, USA). After blocking with 5% skim milk for 2 h at room temperature, the membranes were incubated overnight at 4 °C with primary antibodies from Abclonal (Wuhan, China): anti-G3BP2 (1:1000), anti-GAPDH (1:5000), anti-IBV-N (1:8000), and anti-FLAG (1:8000). Subsequently, the membranes were washed with TBST and incubated with an HRP-conjugated secondary antibody (1:5000) for 1.5 h at room temperature. Protein bands were visualized using a chemiluminescence kit (Thermo Fisher Scientific, Waltham, MA, USA) and quantified with ImageJ software (version 1.53e; National Institutes of Health, Bethesda, MD, USA).

### 2.9. Statistical Analysis

All experimental data were analyzed using GraphPad Prism 8 software (GraphPad Software, San Diego, CA, USA) and are presented as the mean ± standard error of the mean (SEM) from three independent experiments. For comparisons against a single control group, we used one-way ANOVA followed by Dunnett’s test. For datasets involving two independent variables (e.g., treatment and time), two-way ANOVA was applied, followed by Šidák’s test for planned pairwise comparisons or Dunnett’s test for multi-group comparisons against a control at each time point. A *p*-value ≤ 0.05 was considered statistically significant.

## 3. Results

### 3.1. IBV-lncRNA Promoted IBV Growth in H1299 Cells

**Figure 1:**
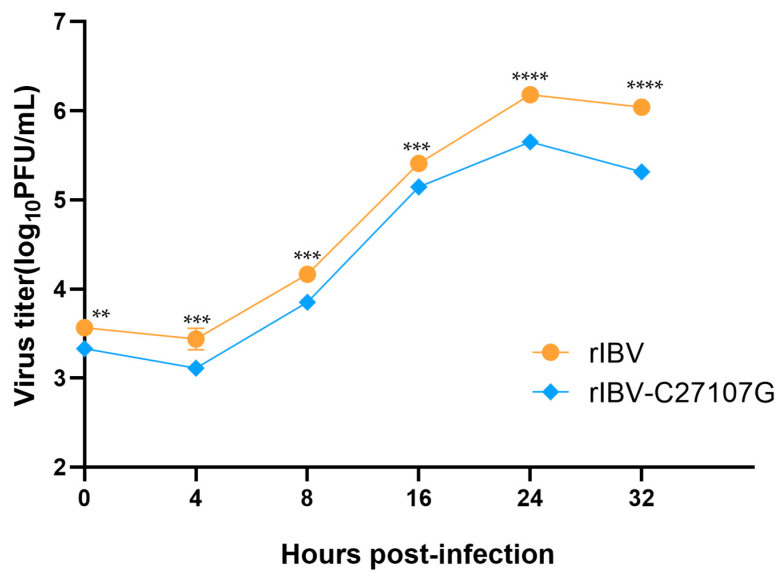
Growth kinetics of rIBV and rIBV-C27107G in H1299 cells. H1299 cells were infected with rIBV or rIBV-C27107G at an MOI of 0.5. Supernatants were collected at the indicated time points post-infection, and viral titers were determined by plaque assay on Vero cells. Data are presented as mean ± SEM. Significant differences are indicated by asterisks (** *p* < 0.01; *** *p* < 0.001; **** *p* < 0.0001), as determined by two-way ANOVA with Šidák’s multiple comparisons test.

H1299 cells were infected with rIBV and rIBV-C27107G at an equal multiplicity of infection (MOI), and supernatants were collected at various time points post-infection for titer determination to plot the viral growth curves (Figure 1). The titers of both viruses in H1299 cells increased over time, showing similar trends at all time points. However, the titer of rIBV was significantly higher than that of rIBV-C27107G at each corresponding time point. The viral titers peaked at 24 h post-infection, with the titer (log_10_ PFU/mL) reaching approximately 6.17 for rIBV and 5.65 for rIBV-C27107G, representing an approximately 5-fold difference. These results suggest that IBV-lncRNA may promote IBV replication in H1299 cells.

### 3.2. Identification of Host Proteins Interacting with IBV-lncRNA by RNA Pull-Down Assay Combined with Mass Spectrometry

**Figure 2:**
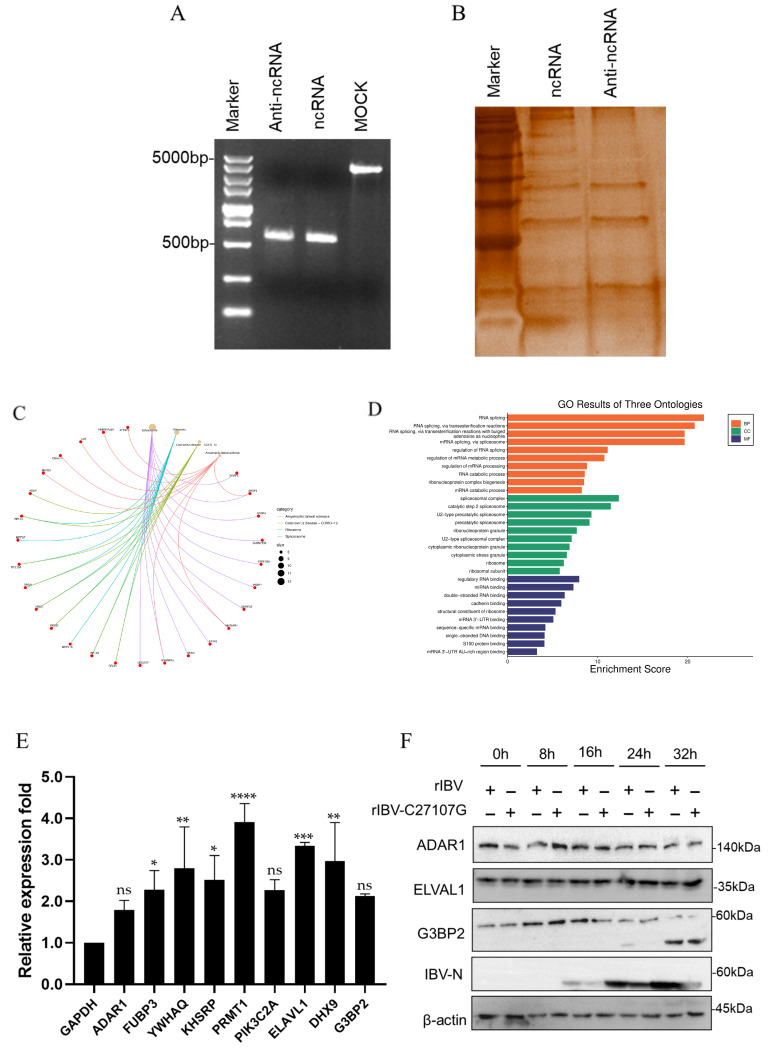
Identification of host proteins interacting with IBV-lncRNA by RNA pull-down coupled with LC-MS/MS. (**A**) The synthesis of biotin-labeled IBV-lncRNA via T7 in vitro transcription. (**B**) Silver-stained SDS-PAGE gel showing proteins pulled down by biotinylated IBV-lncRNA (ncRNA) or an antisense RNA control (Anti-ncRNA). (**C**,**D**) GO term enrichment analysis (**C**) and KEGG pathway analysis (**D**) of the candidate host proteins identified by mass spectrometry. (**E**) mRNA expression levels of nine selected candidate interaction proteins in H1299 cells infected with rIBV, as determined by RT-qPCR. (**F**) Western blot analysis of ADAR1, ELAVL1, and G3BP2 protein expression in H1299 cells at different time points post-infection with rIBV. Significant differences are indicated by asterisks (ns, not significant; * *p* < 0.05; ** *p* < 0.01; *** *p* < 0.001; **** *p* < 0.0001), as determined by one-way ANOVA with Dunnett’s test.

To identify host proteins interacting with IBV-lncRNA, we synthesized biotin-labeled IBV-lncRNA via T7 in vitro transcription (Figure 2A). An RNA pull-down assay combined with LC-MS/MS was used to screen H1299 cell lysates for specific binding partners. Silver staining of SDS-PAGE gels showed distinct protein bands in the IBV-lncRNA group compared to the anti-IBV-lncRNA control group (Figure 2B). LC-MS/MS analysis identified 115 candidate binding proteins.

Gene Ontology (GO) enrichment analysis revealed that these proteins were mainly localized in the cytoplasm and organelles, and were involved in mRNA splicing, RNA metabolism, and ribonucleoprotein complex assembly. Kyoto Encyclopedia of Genes and Genomes (KEGG) pathway analysis indicated enrichment in spliceosome, metabolic pathways, and COVID-19-related terms (Figure 2C,D).

Based on their reported roles in antiviral immunity, nine candidate proteins, ADAR1 (Adenosine deaminase acting on RNA 1), FUBP3 (Far-upstream element-binding protein 3), YWHAQ (tyrosine 3-monooxygenase/tryptophan 5-monooxygenase activation protein, theta polypeptide), KHSRP (KH-type splicing regulatory protein), PRMT1 (Protein arginine methyltransferase 1), PIK3C2A (Phosphatidylinositol-4-phosphate 3-kinase catalytic subunit type 2 alpha), ELAVL1 (ELAV like RNA binding protein 1), DHX9 (DExH-box helicase 9), and G3BP2 were selected for further validation. RT-qPCR confirmed that their mRNA levels were significantly upregulated in rIBV-infected H1299 cells (Figure 2E). Western blot analysis of ADAR1, ELAVL1, and G3BP2 showed that only G3BP2 exhibited decreased expression and cleavage fragments at mid-to-late infection stages (Figure 2F). Given its dynamic expression changes and established role in virus–host interactions, G3BP2 was chosen for subsequent functional studies.

### 3.3. Effect of G3BP2 on Viral Replication

**Figure 3:**
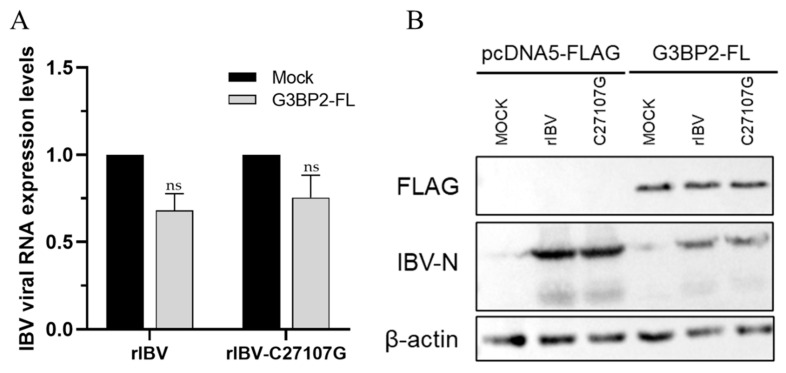
Overexpression of G3BP2 inhibits IBV replication. H1299 cells were transfected with pcDNA5-G3BP2-FL or empty vector for 24 h, followed by infection with rIBV or rIBV-C27107G (MOI = 0.5). (**A**) Viral RNA levels were quantified by qPCR at 24 h post-infection. (**B**) Viral N protein expression was detected by Western blot at 24 h post-infection. Significant differences are indicated by asterisks (ns, not significant), as determined by two-way ANOVA with Šidák’s multiple comparisons test.

To determine the role of G3BP2 in IBV replication, we overexpressed or knocked down G3BP2 in H1299 cells using RNA interference (RNAi), followed by infection with rIBV or rIBV-C27107G. For overexpression studies, the coding sequence of G3BP2 was cloned into a FLAG-tagged pcDNA5 vector to generate the plasmid pcDNA5-G3BP2-FL. H1299 cells were transfected with this construct or an empty vector control. At 24 h post-transfection, cells were infected with rIBV or rIBV-C27107G, and samples were collected 24 h post-infection. RT-qPCR and Western blot analyses showed that G3BP2 overexpression significantly suppressed viral RNA replication (Figure 3A) and N protein expression (Figure 3B). Although viral RNA levels in rIBV-infected cells were slightly lower than those in the rIBV-C27107G group, the difference was not statistically significant. No significant difference in protein expression was observed either. These results suggest that G3BP2 overexpression inhibits IBV replication, possibly by interfering with viral transcription or genome replication.

**Figure 4:**
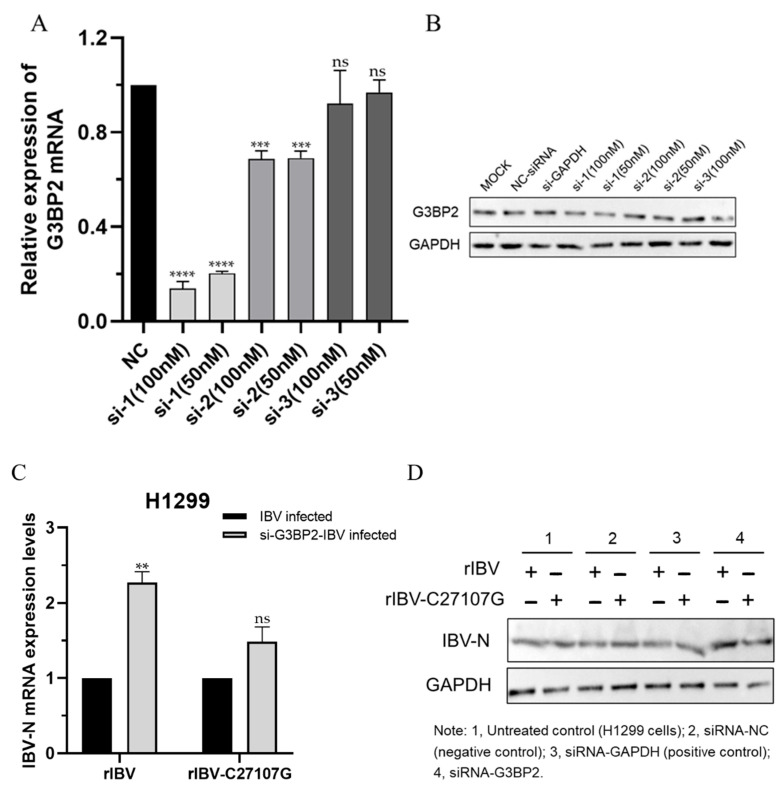
Knockdown of G3BP2 enhances IBV replication. (**A**,**B**) Validation of G3BP2 knockdown efficiency in H1299 cells transfected with three specific siRNAs targeting G3BP2, along with negative control siRNA (NC-siRNA) and GAPDH-targeting siRNA (GAPDH-siRNA). G3BP2 mRNA levels (**A**) and protein expression (**B**) were analyzed by qPCR and Western blot, respectively. (**C**,**D**) Effect of G3BP2 knockdown on viral replication. H1299 cells transfected with siG3BP21 or NCsiRNA were infected with rIBV or rIBV-C27107G (MOI = 0.5). Viral RNA copies (**C**) and N protein expression (**D**) were measured at 24 h post-infection. Significant differences are indicated by asterisks (ns, not significant; ** *p* < 0.01; *** *p* < 0.001; **** *p* < 0.0001), as determined by one-way ANOVA with Dunnett’s test (**A**), and two-way ANOVA with Šidák’s multiple comparisons test (**C**).

Three specific siRNAs targeting G3BP2 were designed and synthesized. A non-targeting siRNA (NC-siRNA) and a GAPDH-targeting siRNA were used as controls. Validation by RT-qPCR and Western blot confirmed that siG3BP2-1 had the highest knockdown efficiency, reducing G3BP2 mRNA levels to approximately 20% of the control (Figure 4A) and substantially decreasing G3BP2 protein expression (Figure 4B). Therefore, siG3BP2-1 was selected for further experiments.

After G3BP2 knockdown, cells were infected with rIBV or rIBV-C27107G. Viral RNA copy numbers increased by 2.2-fold in the rIBV group and 1.5-fold in the rIBV-C27107G group compared to controls (Figure 4C). Western blot analysis further revealed that G3BP2 knockdown significantly enhanced viral N protein expression, with a more pronounced effect in rIBV-infected cells than in the rIBV-C27107G group (Figure 4D).

These results indicate that knockdown of G3BP2 promotes IBV replication, and this enhancing effect is stronger in the presence of IBV-lncRNA (i.e., in rIBV-infected cells).

### 3.4. The Interaction Between IBV-lncRNA and G3BP2

**Figure 5:**
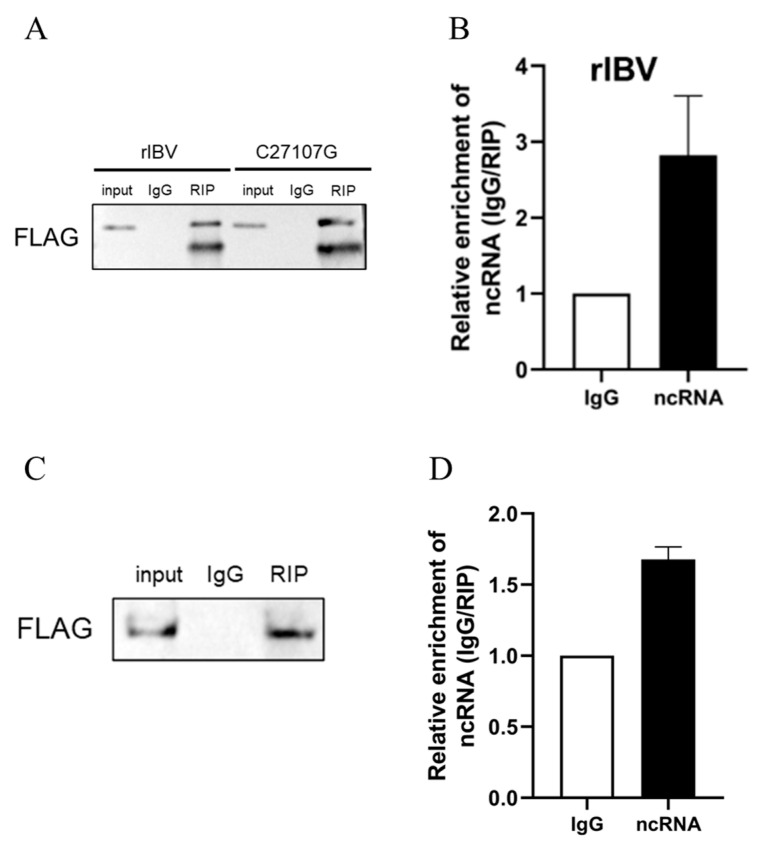
Validation of the interaction between G3BP2 and IBV-lncRNA by RNA immunoprecipitation. (**A**) Western blot analysis of G3BP2 immunoprecipitation using an anti-FLAG antibody in H1299 cells expressing FLAG-tagged G3BP2 after infection with rIBV or rIBV-C27107G. IgG was used as a control for non-specific binding. (**B**) RT-qPCR quantification of IBV-lncRNA enriched in the immunoprecipitates from (**A**). (**C**) Western blot analysis of IBV-lncRNA enrichment in H1299 cells co-transfected with G3BP2 and ncRNA expression plasmids in an overexpression system. (**D**) RT-qPCR quantification of IBV-lncRNA enriched in the immunoprecipitates from (**C**).

To confirm the specific interaction between G3BP2 and IBV-lncRNA, H1299 cells expressing FLAG-tagged G3BP2 were infected with either rIBV or the mutant rIBV-C27107G. Western blot analysis verified successful immunoprecipitation of G3BP2 using an anti-FLAG antibody, with no detectable signal in the IgG control (Figure 5A). RT-qPCR revealed significant enrichment of ncRNA in immunoprecipitate from rIBV-infected cells, but not in those infected with rIBV-C27107G (Figure 5B). In a complementary overexpression assay, co-transfection of G3BP2 and ncRNA expression plasmids also resulted in marked enrichment of ncRNA (Figure 5C,D). Together, these results demonstrate that G3BP2 specifically binds to IBV-lncRNA under both viral infection and overexpression conditions.

### 3.5. Viral Infection Affects the Expression of Novel-340 in H1299 Cells

**Figure 6:**
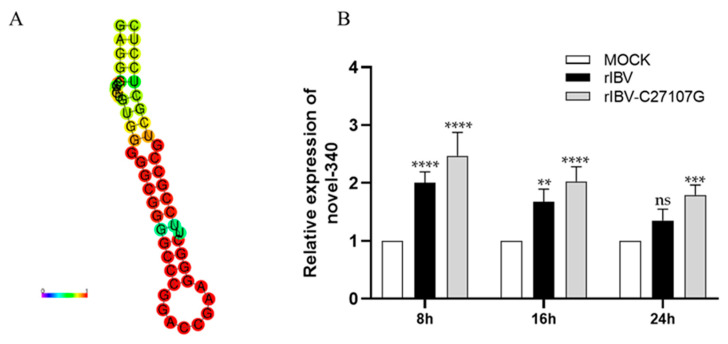
Prediction and expression analysis of novel-340. (**A**) Predicted secondary structure of the novel miRNA precursor novel-340. The secondary structure was generated using RNAfold software. (**B**) Expression dynamics of novel-340 during viral infection. H1299 cells were infected with rIBV or rIBV-C27107G (MOI = 0.5). The expression levels of novel-340 at different time points post-infection were quantified by stem-loop RT-qPCR. U6 snRNA was used as an internal control. Significant differences are indicated by asterisks (ns, not significant; ** *p* < 0.01; *** *p* < 0.001; **** *p* < 0.0001), as determined by two-way ANOVA with Dunnett’s test.

Based on previous small RNA sequencing data, we identified a previously unannotated miRNA, novel-340, which is highly expressed in H1299 cells. To examine the effect of viral infection on its expression, we further validated and characterized this miRNA. The precursor sequence of novel-340 was predicted by RNAfold web server (accessed on 15 July 2025 from website: http://rna.tbi.univie.ac.at/cgi-bin/RNAWebSuite/RNAfold.cgi) to form a typical stem-loop structure (Figure 6A). The mature miRNA is 22 nt in length (5′-CUUCCGCCUCCCGUCGCUCCUC-3′) and shares no significant homology with known miRNAs, supporting its classification as a novel miRNA.

Stem-loop RT-qPCR was used to assess the expression dynamics of novel-340 during viral infection. The results indicated that, while IBV infection led to an overall upregulation of novel-340 compared to mock-infected cells, its expression gradually decreased over the time course of rIBV infection. Moreover, expression levels in rIBV-infected cells were consistently lower than those in cells infected with rIBV-C27107G (Figure 6B), consistent with the earlier sequencing data. These findings suggest that IBV-lncRNA suppresses the expression of novel-340.

### 3.6. Effect of Novel-340 on Viral Replication

**Figure 7:**
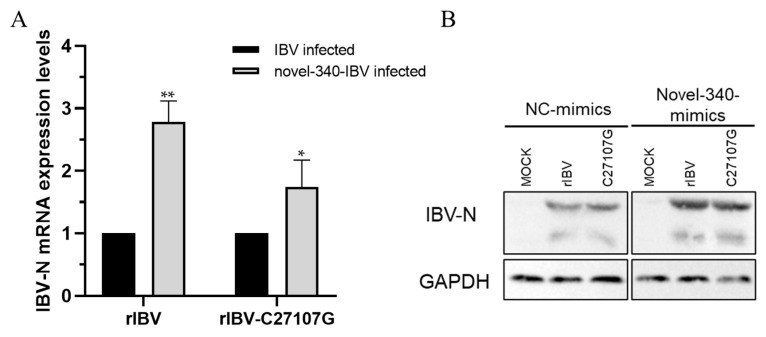
Overexpression of novel-340 promotes IBV replication. H1299 cells were transfected with a novel-340 mimic or negative control (NC-mimics), followed by infection with rIBV or rIBV-C27107G (MOI = 0.5). Viral RNA levels (**A**) and N protein expression (**B**) were analyzed by RT-qPCR and Western blot, respectively, at 24 h post-infection. GAPDH served as a loading control. Significant differences are indicated by asterisks (* *p* < 0.05; ** *p* < 0.01), as determined by two-way ANOVA with Šidák’s multiple comparisons test.

To investigate the function of novel-340 during IBV infection, novel-340 was overexpressed in H1299 cells, followed by infection with either rIBV or rIBV-C27107G. RT-qPCR and Western blot results demonstrated that overexpression of novel-340 significantly increased viral RNA levels and N protein expression, with a more pronounced promoting effect observed for the wild-type rIBV (Figure 7). These results indicate that novel-340 enhances IBV replication and exhibits a more significant regulatory effect on the wild-type virus.

### 3.7. Novel-340 Targets the G3BP2 Gene

**Figure 8:**
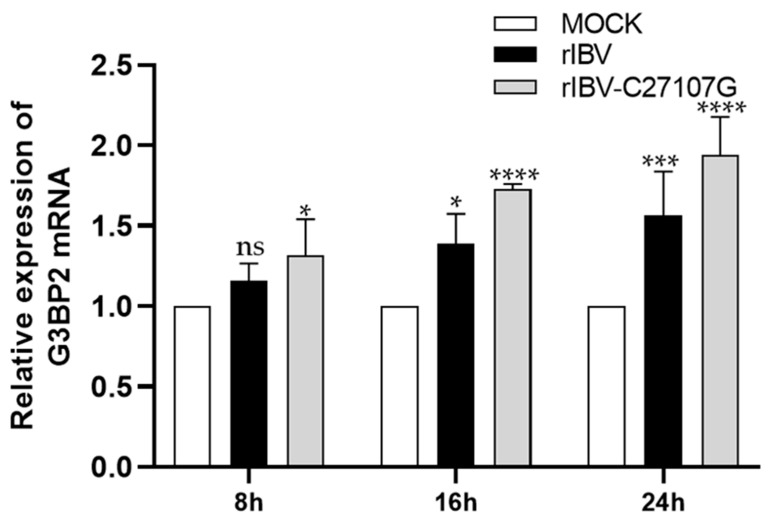
Expression dynamics of G3BP2 during viral infection. H1299 cells were infected with rIBV or rIBV-C27107G (MOI = 0.5). The expression levels of G3BP2 at different time points post-infection were quantified by RT-qPCR. GAPDH was used as an internal control. Significant differences are indicated by asterisks (ns, not significant; * *p* < 0.05; *** *p* < 0.001; **** *p* < 0.0001), as determined by two-way ANOVA with Dunnett’s test.

G3BP2, a core component of stress granules, participates in multiple cellular processes. Previous studies have demonstrated that IBV infection leads to a gradual increase in G3BP2 expression in H1299 cells, whereas the expression of novel-340 shows a declining trend. In cells infected with the ncRNA-deficient strain rIBV-C27107G, G3BP2 expression was higher than in wild-type rIBV-infected cells, exhibiting a negative correlation with the elevated expression pattern of novel-340 (Figure 6B and Figure 8). The specific interaction between IBV-lncRNA and G3BP2 was further confirmed by RNA pull-down combined with mass spectrometry and RNA immunoprecipitation assays.

**Figure 9:**
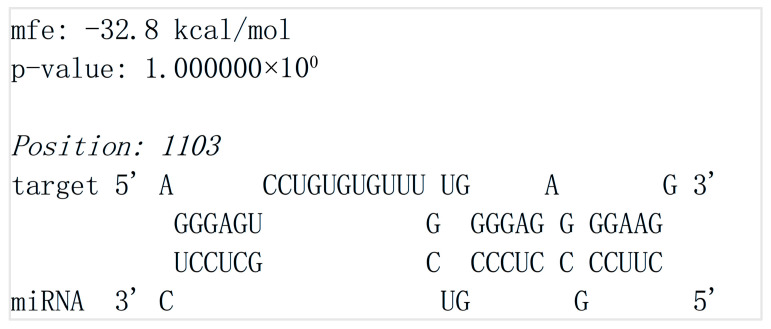
Prediction of novel-340 binding to the 3′-UTR of G3BP2. Predicted secondary structure of the novel-340 and G3BP2 3′-UTR interaction, as generated by RNAhybrid software. The calculated free energy of the interaction is −32.8 kcal/mol.

To explore the regulatory relationship between G3BP2 and novel-340, this study utilized the RNAhybrid software to predict the potential binding of novel-340 to the 3′-UTR of G3BP2 (NM_203505.3). The results indicate a putative binding site for novel-340 at nucleotides 1103–1124 of the G3BP2 3′-UTR, with a binding free energy of −32.8 kcal/mol (Figure 9). This thermodynamically favorable interaction is characteristic of miRNA-target gene binding, suggesting that novel-340 may participate in the post-transcriptional regulation of G3BP2 by binding to its 3′-UTR.

**Figure 10:**
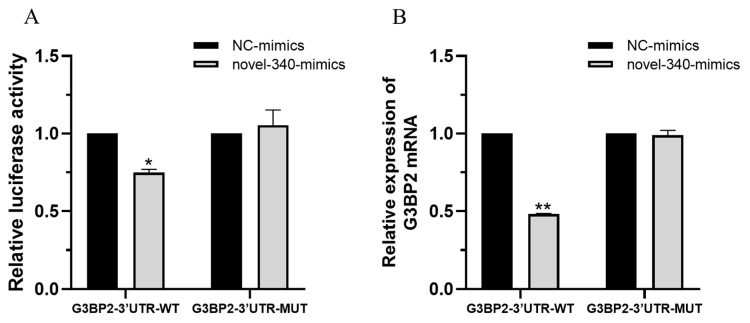
Validation of novel-340 targeting G3BP2 using a dual-luciferase reporter system. (**A**) H1299 cells were co-transfected with wild-type (WT) or mutant (MUT) G3BP2 3′-UTR luciferase reporter plasmids and either novel-340 mimics or NC mimics. Luciferase activity was measured 48 h post-transfection. Firefly luciferase activity was normalized to Renilla luciferase activity. (**B**) G3BP2 mRNA levels originating from the transfected reporter plasmids were quantified by qPCR at 48 h post-transfection. Data in both (**A**,**B**) are presented as mean ± SEM from three independent experiments. * *p* < 0.05, ** *p* < 0.01.

To validate the targeted regulatory relationship between novel-340 and G3BP2, we performed a dual-luciferase reporter assay. H1299 cells were co-transfected with either wild-type (pmirGLO-G3BP2-3′-UTR-WT) or mutant (pmirGLO-G3BP2-3′-UTR-MUT) reporter plasmids, along with novel-340 mimics or negative control (NC) mimics. As shown in Figure 10A, novel-340 mimics significantly reduced the relative luciferase activity in cells transfected with the wild-type reporter plasmid, while no significant effect was observed with the mutant construct. Subsequent qPCR analysis confirmed that novel-340 overexpression specifically reduced G3BP2 mRNA levels by approximately 50% in cells carrying the wild-type reporter plasmid, with no significant effect detected in the mutant group (Figure 10B). These findings demonstrate that novel-340 specifically binds to the 3′-UTR of G3BP2 and functions as a negative regulator of its expression.

**Figure 11:**
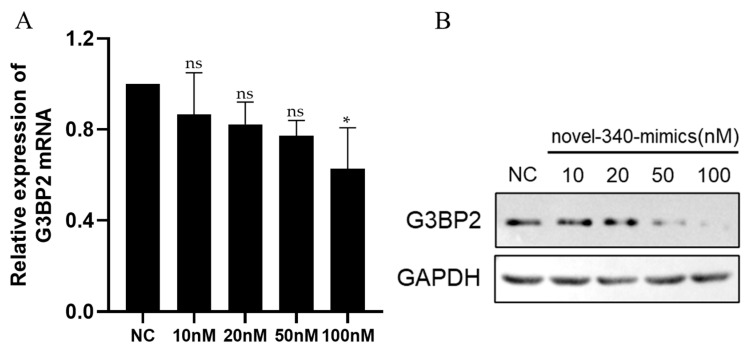
Dose-dependent inhibition of G3BP2 expression by novel-340. H1299 cells were transfected with increasing concentrations of novel-340 mimics or a constant concentration of NC mimic. (**A**) G3BP2 mRNA levels were analyzed by qPCR 48 h post-transfection. (**B**) G3BP2 protein levels were analyzed by Western blot at 48 h post-transfection. GAPDH served as a loading control. Significant differences are indicated by asterisks (ns, not significant; * *p* < 0.05), as determined by one-way ANOVA with Dunnett’s test.

To investigate the regulatory role of novel-340 on G3BP2, H1299 cells were transfected with escalating doses of novel-340 mimics. Changes in G3BP2 expression at both mRNA and protein levels were examined by qPCR and Western blot analysis, respectively. The results revealed a clear dose-dependent decrease in G3BP2 mRNA and protein expression with increasing concentrations of novel-340 (Figure 11), demonstrating that novel-340 suppresses G3BP2 expression at the post-transcriptional level.

Collectively with the dual-luciferase reporter assay findings, these results confirm that novel-340 mediates suppression of G3BP2 expression through direct targeting of its 3′-UTR, thereby providing crucial experimental evidence for understanding its regulatory mechanism during IBV infection.

## 4. Discussion

Our previous investigations have demonstrated that IBV-lncRNA exerts no significant influence on virus replication and pathogenicity in Vero cells [13]. Nevertheless, in the present study, IBV-lncRNA exhibited a phenomenon of facilitating the replication of IBV in H1299 cells. The cause of this difference might be that Vero cells belong to a cell line with a defective interferon system, whereas H1299 cells can express interferons in a normal manner. This also implies that IBV-lncRNA has the function of regulating the host’s immune response. In summary, these results accomplished our goal of demonstrating the immune-regulatory function of IBV-lncRNA and its critical impact on viral replication.

To identify host proteins that interact with IBV-lncRNA, we first performed an RNA pull-down assay followed by mass spectrometry analysis. This screen identified G3BP2 as a primary candidate (Figure 2). We then confirmed the specific nature of this interaction using an independent method, RNA immunoprecipitation (RIP), which demonstrated direct binding between IBV-lncRNA and G3BP2 (Figure 5). It is noteworthy that G3BP2 was prioritized for further investigation from a pool of candidate proteins which were initially selected based on GO/KEGG enrichment analysis for their strong association with viral infection and antiviral immune pathways [14]. The overexpression of G3BP2 significantly inhibited the replication of IBV, while its knockdown promoted the proliferation of IBV. G3BP2 is a member of the G3BP family, which encompasses G3BP1, G3BP2, and two splicing variants of G3BP2, specifically G3BP2a and G3BP2b. G3BP proteins possess similar domain structures, cooperatively regulate the assembly process of stress granules [15]. Prior research has indicated that both G3BP1 and G3BP2 play significant roles in the formation of stress granules (SG) [16]. As a typical RNA-binding protein, G3BP2 is not only a core factor in SG assembly but also acts as a target for multiple RNA viruses (e.g., dengue virus DENV and Zika virus ZIKV) to disrupt the host’s antiviral response or assist in the construction of the viral replication complex [17]. Owing to their multiple functions in RNA stability, translation regulation, and stress response, G3BP proteins have emerged as one of the primary targets for viral regulation [14]. During the infection of severe acute respiratory syndrome coronavirus 2 (SARS-CoV-2), G3BP1 and G3BP2 play a vital role in regulating the interaction between the host and the virus, and their functions in cellular defense are diverse, not confined to the stress granule pathway [15]. During viral infection, stress granules act as storage locations for mRNA and engage in the antiviral activities of host cells to restrict virus dissemination [18]. Research indicates that the IBV nsp15 protein (ribonuclease) can impede the formation of eIF2α-dependent and eIF2α-independent stress granules (SGs), counteract the host’s antiviral response, and thus sustain efficient virus replication among coronaviruses of diverse genera [19]. Innate immune signaling molecules, including PKR, MDA5, TLR3, and MAVS, can co-localize with GTPase-activating protein-binding protein 1 (G3BP stress granule assembly factor 1, G3BP1) granules [17]. Subsequent investigations further verify that the nsp15 proteins from porcine epidemic diarrhea virus (PEDV), porcine transmissible gastroenteritis virus (TGEV), SARS-CoV, and SARS-CoV-2 possess a conserved function in interfering with the formation of chemically induced stress granules [20]. Therefore, it is highly probable that coronaviruses inhibit the assembly of host antiviral stress granules via a similar mechanism mediated by nsp15, thereby synergistically enhancing virus replication efficiency.

In the late phase of IBV infection, the level of G3BP2 protein declines, and cleavage bands emerge concurrently. This expression pattern exhibits a high degree of consistency with other virus-related reports. For instance, PEDV triggers the cleavage of G3BP1 protein by activating Caspase-8, thereby disrupting stress granules and facilitating virus replication [21]. In the simian hemorrhagic fever virus (SHFV) infection model, the generation of specific cleavage fragments of G3BP1 (58 kDa) and G3BP2 (50 kDa) mediated by viral proteases was also noted [22]. Based on the aforementioned findings, it is hypothesized that IBV may regulate the stability of G3BP2 via a conserved proteolytic mechanism, collaborate with IBV-lncRNA to impede the assembly of stress granules, and jointly foster the maturation and release of virus particles.

Viral infection can specifically alter host microRNA (miRNA) expression to create a cellular environment favorable for viral replication. Conversely, these changes may also reflect the host’s innate immune response, such as miRNA-mediated antiviral effects that suppress replication. Sardar et al. [23] identified 2197 host miRNAs targeting genes interacting with SARS-CoV-2 proteins; these miRNAs regulate T-cell differentiation and activation, viral replication, and immune responses. Host miR-122 binds the 5′-UTR of the hepatitis C virus (HCV) genome [24], exerting dual functions: it inhibits viral translation (antiviral) yet stabilizes viral RNA to promote replication (proviral)—illustrating miRNAs’ dynamic, context-dependent roles during infection. Beyond host miRNAs, virus-induced miRNA dysregulation serves both as a key regulatory node in host immunity and as a viral strategy for persistent infection. For example, hepatitis B virus-associated hepatocellular carcinoma (HBV-HCC) involves coordinated dysregulation of host genes and miRNAs, enabling a balance between viral replication and immune evasion [25].

In this study, we identified a novel host miRNA (i.e., novel-340) in IBV-infected H1299 cells by small RNA sequencing analysis. Throughout the course of IBV infection, the expression level of novel-340 declined significantly as the infection duration increased. The finding that novel-340 expression is higher following IBV-lncRNA disruption points to a repressive role of IBV-lncRNA on novel-340. The subsequent demonstration that novel-340 overexpression enhances viral replication indicates it is a negative regulator of infection, an effect we attribute to its direct targeting and downregulation of G3BP2. Therefore, IBV-lncRNA promotes infection by suppressing novel-340 and its inhibitory effect on G3BP2. Conversely, the expression level of G3BP2 demonstrated an upward tendency over time, and at the same time point, the expression level of G3BP2 in the rIBV-C27107G group was higher than that in the rIBV group. This suggests that IBV-lncRNA may alleviate the translational inhibitory effect of novel-340 on G3BP2 by suppressing novel-340, thereby attaining a dynamic equilibrium between stress granule assembly and the virus replication requirement. This regulatory network exhibits a high degree of consistency with the classic mechanism and function of miRNA, suggesting that there might exist a targeting relationship between novel -340 and G3BP2. IBV may establish an IBV-lncRNA/novel-340/G3BP2 interaction network by modulating the expression of host miRNA. This network collaboratively participates in suppressing the antiviral activity of G3BP2, thereby creating a favorable environment for virus replication.

In conclusion, our research indicates that IBV-lncRNA can directly interact with G3BP2 and potentially interfere with the stress granule assembly process mediated by it, which facilitates the virus’s immune evasion. Moreover, IBV-lncRNA may also limit the excessive suppression of G3BP2 by novel-340 through down-regulating the expression of novel-340, thereby preventing the imbalance of the immune response caused by the complete loss of the host stress granule function. This discovery has provided evidence for elucidating the complex mechanism of IBV-lncRNA within the virus–host interaction network and has also offered new insights for formulating broad-spectrum antiviral therapies targeting host factors.

To facilitate detailed mechanistic dissection, this study employed a laboratory-adapted IBV strain capable of stable infection in human cell lines [26]. This experimental model was selected for in-depth analysis due to practical considerations: human-specific antibodies and molecular tools are more readily available, whereas chicken-specific reagents remain relatively scarce, substantially limiting the ability to conduct mechanistic validation at comparable depth in avian systems. Despite the species difference, sequence alignment analysis (Figure S1) reveals that human G3BP2 (NP_036429.2) and its chicken homolog (XP_420536) exhibit both divergent and conserved regions. While significant variations occur in the N-terminal nuclear transport factor 2-like domain and central disordered region (potentially affecting species-specific protein interactions) [27], the C-terminal RNA-binding domain shows remarkable conservation, with only a single amino acid difference at position 390 (Ile in human, Val in chicken). This structural conservation strongly suggests that the core mechanism of IBV-lncRNA modulating stress granule formation via G3BP2 is likely operative in avian cells.

Consequently, targeting the G3BP2 stress granule pathway represents a promising host-directed antiviral strategy, potentially offering a higher resistance barrier and broad-spectrum efficacy against diverse IBV strains and related avian coronaviruses. A key limitation of this study is that the proposed mechanism was elucidated in a human lung cell model (H1299), and its physiological relevance in the natural avian host remains to be fully established. To bridge this gap and translate these findings toward practical applications, future work will prioritize validating this mechanism in avian systems. This includes confirming the functional IBV-lncRNA-G3BP2 interaction and its regulatory impact on the miRNA axis in DF-1 cells and primary chicken cells, as well as analyzing expression dynamics in IBV-infected chicken tissues to correlate molecular changes with viral load and pathology. These essential studies will bridge the gap between fundamental mechanisms and veterinary utility, laying a solid foundation for developing host-targeted control strategies against infectious bronchitis.

## 5. Conclusions

In conclusion, we have identified the host protein G3BP2 as a specific binding partner of coronavirus IBV-lncRNA. This discovery opens new avenues for understanding how viral non-coding RNAs interact with the host machinery. Further research is needed to elucidate the functional consequences of this interaction on host gene expression and viral replication.

## Figures and Tables

**Table 1:** Primers used for construction of overexpression plasmid.

Primer	Sequences (5′-3′)
G3BP2-FL-F	cgcggatccATGGTTATGGAGAAGCCCAGT
G3BP2-FL-R	ccgctcgagGCGACGCTGTCCTGTGAA

Note: Lowercase letters within primer sequences denote protective bases and restriction enzyme sites.

**Table 2:** Sequences of novel-340 mimic, siRNA and negative control.

Gene	Sequences (5′-3′)
novel-340-mimics	CUUCCGCCUCCCGUCGCUCCUC
NC-mimics	UUGUACUACACAAAAGUACUG
siRNA-G3BP2-1	GGAGAAGAACUUAGAAGAATT
siRNA-G3BP2-2	GGAGUAGAUGCUAGUGGAATT
siRNA-G3BP2-3	GAGGAGAUAUGGAACAGAATT
siRNA-GAPDH	GUAUGACAACAGCCUCAAGTT
siRNA-NC	UUCUCCGAACGUGUCACGUTT

**Table 3:** Primers used for Dual-Luciferase Reporter Assay System.

Primer	Sequences (5′-3′)
G3BP2-WT-F	ctagctagcATTCCAGTCTTGCTGGTA
G3BP2-WT-R	ccgctcgagGAAGTCCGAATGAACGGT
G3BP2-MUT-F	ctagctagcGTGTTTCACATTCCTGAGGAAGTCAGTTATTTGAGTAAGCCT
G3BP2-MUT-R	ccgctcgagCTCAGGAATGTGAAACACAAACACACAGGACTCCCTCC

Note: Lowercase letters within primer sequences denote protective bases and restriction enzyme sites.

**Table 4:** Primer sequences.

Gene	Primer	Sequences (5′-3′)	Temperature
*IBV-lncRNA*	F	TGGAAACGAACGGTAGACCC	58 °C
	R	TTCCCCTAATGGGCGTCCTA	
*G3BP2*	F	GTGTTTGGTGATTCTGAGCCTG	62 °C
	R	GCACAGGTTCAGGAGATGGTT	
*novel-340*	F	CTTCCGCCTCCCGTCG	63 °C
	R	AGTGCAGGGTCCGAGGTATT	
*IBV-N*	F	TGAAGGTAGCGGTGTTCCTG	62 °C
	R	CCACGGTTCAGGGGAATGAA	
*GAPDH*	F	GTCAAGGCTGAGAACGGGAA	62 °C
	R	AGTGATGGCATGGACTGTGG	
*U6*	F	TTGTGTTTCCTAAATCCAACCATT	63 °C
	R	GCAGTTAACAGCAGATTTTTCAC	

## Data Availability

The original contributions presented in this study are included in the article/Supplementary Material. Further inquiries can be directed to the corresponding author(s).

## Supplementary Materials

The following supporting information can be downloaded at: https://www.mdpi.com/article/10.3390/vetsci13030215/s1, Figure S1: Sequence alignment of human (NP_036429.2) and chicken (XP_420536) G3BP2 proteins.
