# Supplementary figures and images for: Long Non-Coding RNA Encoded by Infectious Bronchitis Virus Facilitates Viral Replication via Direct Interaction with G3BP2 and Expression Regulation of a Novel Host MicroRNA
Source: Vet Sci. 2026 Feb 25;13(3):215. doi: 10.3390/vetsci13030215 (PMC13030203; doi:10.3390/vetsci13030215)

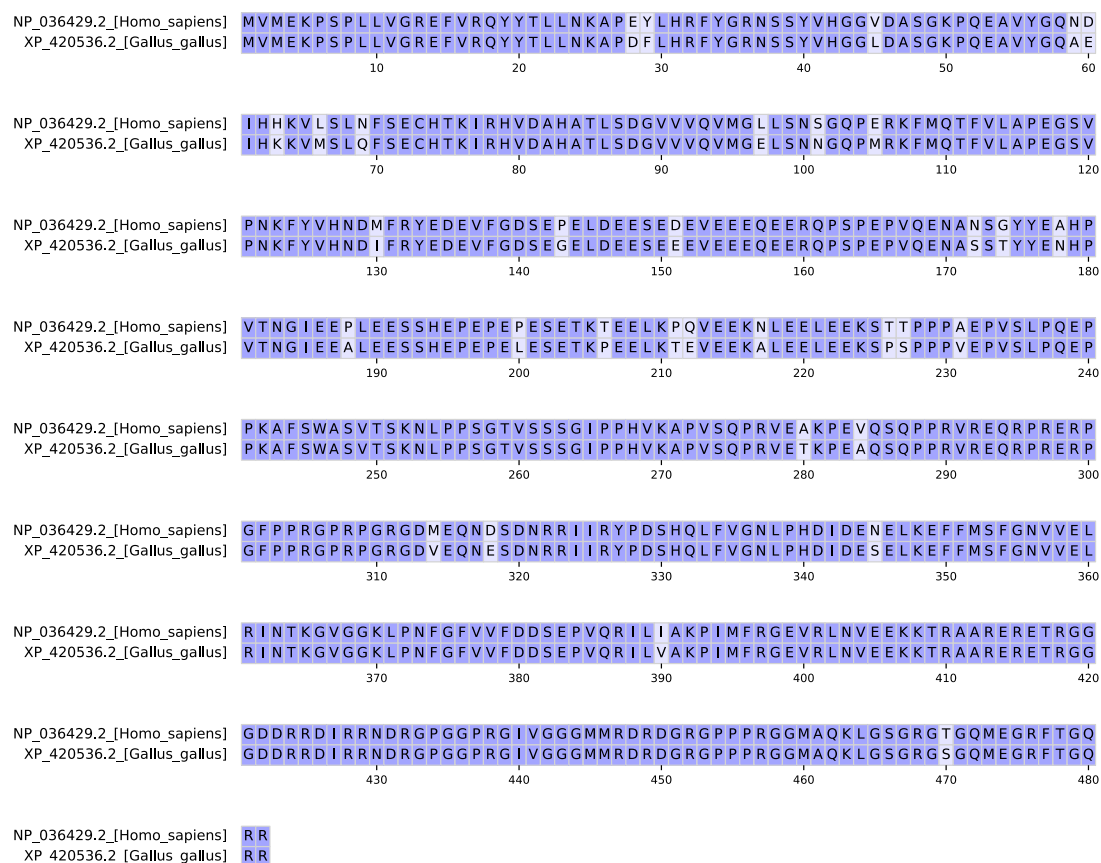

**Figure S1.** Sequence alignment of human (NP\_036429.2) and chicken (XP\_420536) G3BP2 proteins.

Supplement: Supplementary file 1 [file vetsci-13-00215-s001.zip › vetsci-4154374-supplementary.pdf]
